# Flecainide and risk of skin neoplasms: Results of a large nested case–control study in Spain and Denmark

**DOI:** 10.3389/fphar.2022.1002451

**Published:** 2022-12-22

**Authors:** Carlen Reyes, Luz M León-Muñoz, Andrea Pistillo, Sigrún Alba Jóhannesdóttir Schmidt, Kasper Bruun Kristensen, Diana Puente, Ana LLorente-García, Consuelo Huerta-Álvarez, Anton Pottegård, Talita Duarte-Salles

**Affiliations:** ^1^ Fundació Institut Universitari per a la Recerca a L'Atenció Primària de Salut Jordi Gol i Gurina (IDIAPJGol), Barcelona, Spain; ^2^ Delegation for the National Plan on Drugs, Ministry of Health, Madrid, Spain; ^3^ Department of Clinical Epidemiology, Aarhus University Hospital, Aarhus, Denmark; ^4^ Department of Dermatology, Aarhus University Hospital, Aarhus, Denmark; ^5^ Clinical Pharmacology, Pharmacy, and Environmental Medicine, Institute of Public Health, University of Southern Denmark, Odense, Denmark; ^6^ Universitat Autònoma de Barcelona, Bellaterra, Spain; ^7^ Pharmacoepiemiology and Pharmacovigilance Division, Spanish Agency for Medicines and Clinical Devices-AEMPS, Madrid, Spain; ^8^ Department of Public Health & Maternal and Child Health, Faculty of Medicine. Complutense University of Madrid-UCM, Madrid, Spain

**Keywords:** case-control studies, flecainide, skin neoplasm, database, melanoma

## Abstract

**Background:** A previous study in Denmark suggested an increased melanoma risk associated with the use of flecainide.

**Objective:** To study the association between flecainide use and the risk of melanoma and non-melanoma skin cancer in Spain and Denmark.

**Methods:** We conducted a multi-database case–control study in (database/study period) Spain (SIDIAP/2005–2017 and BIFAP/2007–2017) and Denmark (Danish registries/2001–2018). We included incident cases of melanoma or non-melanoma skin cancer (NMSC) aged ≥18 with ≥2 years of previous data (≥10 years for Denmark) before the skin cancer and matched them to controls (10:1 by age and sex). We excluded persons with immunosuppression or previous cancer. We defined ever-use as any prescription fill and high-use as a cumulative dose of at least 200 g (reference: never-use). We categorized a cumulative dose for a dose–response assessment. We used conditional logistic regression to compute ORs (95% CI) adjusted for photosensitizing, anti-neoplastic, disease-specific drugs and comorbidities.

**Results:** The total numbers of melanoma/NMSC cases included were 7,809/64,230 in SIDIAP, 4,661/31,063 in BIFAP, and 27,978/152,821 in Denmark. In Denmark, high-use of flecainide was associated with increased adjusted ORs of skin cancer compared with never-use [melanoma: OR 1.97 (1.38–2.81); NMSC: OR 1.34 (1.15–1.56)]. In Spain, an association between high-use of flecainide and NMSC was also observed [BIFAP: OR 1.42 (1.04–1.93); SIDIAP: OR 1.19 (0.95–1.48)]. There was a non-significant dose–response pattern for melanoma in Denmark and no apparent dose–response pattern for NMSC in any of the three databases. We found similar results for ever-use of flecainide.

**Conclusion:** Flecainide use was associated with an increased risk of melanoma (Denmark only) and NMSC (Denmark and Spain) but without substantial evidence of dose–response patterns. Further studies are needed to assess for possible unmeasured confounders.

## Introduction

The burden of skin cancer, comprising both melanoma and non-melanoma skin cancer (NMSC), has increased in the past decades (Saginala et al., 2021). In 2020, estimates from the global statistics (GLOBOCAN) identified 325,000 new cases of melanoma (1.7% of all cancer diagnoses) and positioned melanoma as the most lethal form of skin cancer in the world (Saginala et al., 2021). In Europe, the skin cancer incidence is marked by a latitudinal north–south gradient. For example, the annual rate of melanoma decreases from 50 per 100,000 in Denmark to 12 per 100,000 in Spain (ECIS, 2022).

The main risk factor for skin cancer is ultraviolet (UV) light exposure, which may explain the increased skin cancer risk and severity found among users of certain photosensitizing drugs (Gonçalo et al., 2011; de Vries et al., 2012; Dika et al., 2021). Among these are certain medications used for cardiovascular diseases such as antihypertensives (Schmidt et al., 2015a; Drucker et al., 2021) and antiarrhythmics (Monk, 1995; Hall et al., 2004; Maoz et al., 2009). In a hypothesis-generating screening study carried out in Denmark, the use of the classic antiarrhythmic flecainide was associated with a 2-fold increased risk of melanoma (Pottegård et al., 2016), with some evidence of a dose–response pattern.

Detailed and up-to-date data on the use of flecainide and its relation with skin cancer risk, including both melanoma and NMSC, are not available from Denmark nor from populations in South Europe. Framing skin cancer as a possible adverse effect of flecainide would allow prescribers to reinforce the preventive measures to treated patients. Therefore, the aim of this study was to assess the association between the use of flecainide and the incidence of melanoma and NMSC in adults from Denmark and Spain.

## Materials and methods

### Design

We conducted a nested case–control study, in three databases of two countries, namely the Danish nationwide health registries in Denmark and the SIDIAP and the BIFAP databases in Spain.

### Data sources

In Denmark, we analyzed data gathered from five nationwide registries: the Danish Cancer Registry (Gjerstorff, 2011), the National Prescription Registry (Pottegård et al., 2017), the National Patient Registry (Schmidt et al., 2015), registries on educational level from Statistics Denmark (Jensen and Rasmussen, 2011), and the Civil Registration System (Schmidt et al., 2014). Linkage was achieved using the personal identification number, which is a unique identifier assigned to all Danish residents since 1968 (Pedersen, 2011). Virtually, all medical care in Denmark is supplied by the national health authorities, allowing true population-based register linkage studies covering all legal residents of Denmark.

In Spain, two databases were used: The SIDIAP (*Sistema d’Informació per al Desenvolupament de la Investigació en Atenció Primària*) and the BIFAP databases (*Base de Datos para la Investigación Farmacoepidemiológica en Atención Primaria*).

The SIDIAP is a large population-based database that gathers routinely collected anonymized healthcare records from nearly six million people (∼75% of the Catalan population) since 2005 (Recalde et al., 2022). Data include sociodemographic, lifestyle and anthropometric information, clinical diagnosis [International Classification of Diseases 10th revision (ICD-10)], laboratory test results, prescribed and dispensed medications, and hospital referrals, among others.

The BIFAP is a longitudinal population-based database that gathers electronic healthcare anonymized information recorded by general practitioners (GPs) and pediatricians belonging to the National Health System throughout Spain (http://bifap.aemps.es/) since 2001. Nine autonomous regions of Spain currently collaborate in this database providing information on lifestyle factors, clinical events, laboratory test results, specialist referrals, electronic prescriptions, and their dispensations in pharmacies. The BIFAP population is representative of the Spanish population in terms of age and sex. At the time of the study, the database covered a total of 7.8 million patients which represents 49 million person-years of follow-up. The mean follow-up of patients in the database was 6.3 years. The BIFAP database has been extensively described elsewhere (Maciá-Martínez et al., 2020).

### Study population, selection of skin cancer cases, and population control

We included all individuals aged between 18 and 85 years old with an incident diagnosis of melanoma or NMSC between 1 January 2001 and 31 of December 2018, in the Danish Cancer Registry database, between the 1 January 2007 and the 31 December 2017 in the BIFAP database, and between 1 January 2006 and the 31 December 2017 in the SIDIAP database. To ensure sufficient follow-up, we required individuals to be registered for at least two consecutive years in the Spanish databases or to be alive at least 10 years prior to the index data for the Danish registries. Given that immunosuppression may increase the risk of skin cancer (International Agency for Research on Cancer (IARC), 2012), we excluded patients with an HIV diagnosis, an organ transplant (Danish population), and dispensation of azathioprine, cyclosporin, or mycophenolate mofetil. We excluded cases and controls with any previous cancer, except for those with previous NMSC in analyses of melanoma as an outcome (only in Denmark).

In Denmark, we included cases with a histologically verified skin cancer diagnosis in the Danish Cancer Registry using the ICD-10 codes. In SIDIAP, we identified cases through ICD-10-Clinical Modification codes linked to two regional population cancer registries available in Catalonia. In BIFAP, all skin cancer diagnoses were validated using an independent validation process which used a specific algorithm and a natural language processing procedure taking into account comments in the free text to validate the potential cases. All the ATC and ICD codes used to identify the variables are listed in Supplementary Table 1.

We selected controls with risk-set sampling in each database. For each case, we matched to up to 10 controls with the same sex and birth year (± 1 for the Spanish databases). We assigned controls on the same index date as the case to whom they were matched. Subjects were eligible for sampling as controls more than one time before possibly becoming cases. Thereby, the calculated odds ratios (ORs) are direct estimates of the incidence rate ratios (IRRs) that would have emerged from a cohort study in the source population.

### Definition of the exposure to flecainide

We defined ever-use of flecainide as having filled at least one dispensation of flecainide (in BIFAP, we used dispensations unless these were not available in which case prescriptions were selected) before the index date. High-use of flecainide was defined as a cumulative dose of at least 200 g of flecainide (1,000 DDD (WHO, 2022)). For the dose–response analyses, we used the following pre-specified categories: ≤19 g, 20–99 g, 100–199 g, 200–399 g, and ≥400 g. In all exposure calculations, we discarded dispensations redeemed during the 2 years before the index date, as recent exposure to flecainide is unlikely to affect skin cancer development and in order to avoid reverse causation (Pottegård and Hallas, 2017).

### Other variables and potential confounders

The list of confounders included comorbid conditions such as diabetes, chronic obstructive pulmonary disease, myocardial infarction (BIFAP and the Danish registries), chronic kidney disease, and alcohol-related disorders (Danish registries), the Charlson Comorbidity Index derived from the prevalence of 14 chronic conditions (SIDIAP and the Danish registries) and related disease-specific drugs, as well as those drugs identified as having photosensitizing properties [retinoids, photosensitizing antibiotics including tetracycline, macrolides, and aminoquinolines, hydrochlorothiazide (Danish registries), amiodarone, and methoxypsoralen/PUVA therapy (Robinson et al., 2013; Schmidt et al., 2015a; Gallelli et al., 2022)] or anti-neoplastic effects [aspirin, NSAIDs, oral corticosteroids, and statins (Friis et al., 2015)]. We defined drug exposure as at least two prescriptions on separate dates, and the comorbid conditions were selected if registered as primary or secondary discharge or outpatient diagnoses. Other confounders gathered included smoking habits (never, current, and former smokers; only available in SIDIAP and BIFAP) and socioeconomic status defined as the highest education level achieved [basic (10 years), medium (11–13 years), higher (>13 years), or unknown] in the Danish registries or the MEDEA deprivation index in SIDIAP (categorized in quintiles U1 for the least deprived and U5 for the most deprived) (Domínguez-Berjón et al., 2008). For drugs and comorbid conditions, information within 2 years prior to the index date was disregarded.

### Statistical analysis

All analyses followed a conventional matched case–control approach. We computed the frequency and proportion of cases and controls within categories of both flecainide exposure and covariates. We used conditional logistic regression analysis to compute odds ratios (ORs) with 95% confidence intervals (CIs) for the association of NMSC or melanoma with flecainide use adjusted for the predefined potential confounders. In addition to the predefined categories of cumulative use, we carried out a formal dose–response test by restricting to flecainide ever-users and estimating the incremental OR for each 100 g of flecainide using logistic regression while also adjusting for sex, age, and index year. In all analyses, we analyzed NMSC and melanoma separately and never-use of flecainide was the reference group unless stated otherwise. We also calculated adjusted OR according to age (<50, 50–59, 60–74, and 75 +) and sex categories with ever-use as exposure for the Spanish databases and with high-use as exposure for the Danish databases. We performed all the analyses locally and for each database separately. We performed analyses in Stata Release 14.2 (StataCorp, College Station, TX, United States) for the BIFAP database, Stata Release 17.0 (StataCorp, College Station, TX, United States) for the Danish registries, and R version 4.0.3 for SIDIAP.

The study was approved by the Danish Data Protection Agency. According to the Danish law, studies based solely on registered data do not require approval from an ethics review board (Thygesen et al., 2011). We obtained approval for all observational research using SIDIAP data from a local ethics committee (Clinical Research Ethics Committee of the IDIAP Jordi Gol) and for the study in BIFAP from the BIFAP Scientific Committee (Ref #01-2018).

## Results

The final population included in the study is reported in the Supplementary Figures S1 and S2 (for the Danish and Spanish population, respectively). Baseline characteristics of the cases and controls with and without melanoma and NMSC are reported in Table 1. A total of 4,661, 7,809, and 27,978 cases of melanoma and 31,063, 64,230, and 152,821 cases of NMSC and their matched controls were included in the BIFAP, SIDIAP, and Danish registries, respectively. Compared with patients with melanoma, those with NMSC were on average 8–12 years older, were more likely to be men (48% vs. 44%–45%), and had a higher comorbidity burden. The vast majority of patients analyzed had never used flecainide (∼99% of cases); however, among those who did, flecainide was more frequently prescribed among NMSC than melanoma in BIFAP (0.84% vs. 0.60%) and SIDIAP (0.79% vs. 0.50%) and at higher cumulative dosages.

**TABLE 1:** Characteristics of melanoma and non-melanoma skin cancer cases and matched controls in BIFAP, SIDIAP, and Danish databases.

	BIFAP		BIFAP		SIDIAP		SIDIAP		Danish databases^1^		Danish Databases^1^	
Subgroup	Malignant melanoma		Non-melanoma skin cancer		Malignant melanoma		Non-melanoma skin cancer		Malignant melanoma		Non-melanoma skin cancer	
	Cases, *N*= 4,661	Controls, *N*= 46,610	Cases, *N* = 31,063	Controls, *N* = 310,630	Cases, *N* = 7,809	Controls, *N* = 75,699	Cases, *N* = 64,230	Controls, *N* = 628,767	Cases, N= 27,978	Controls, *N* = 279,780	Cases, *N* = 152,821	Controls, *N* = 1,528,210
Age, median (interquartile range)	61 (48–73)	61 (48–73)	73 (63–82)	73 (63–82)	59.0 (46–70)	59 (46–70)	71 (61–78)	71 (61–78)	59 (46–70)	59 (46–70)	67 (57–75)	67 (57–75)
Male, sex, n (%)	2,058 (44.1)	20,580 (44.1)	15,025 (48.4)	150,250 (48.4)	3,441 (44.1)	33,425 (44.2)	30,819 (48.0)	302,272 (48.1)	12,841 (45.9)	128,410 (45.9)	74,500 (48.7)	745,000 (48.7)
Use of photosensitizing drugs, n (%)												
Topical retinoids	0 (0.0)	0 (0.0)	<5 (0.0)	13 (0.0)	NA	NA	NA	NA	202 (0.7)	1,638 (0.6)	520 (0.3)	3,628 (0.2)
Oral retinoids	<5 (0.1)	20 (0.0)	12 (0.0)	75 (0.0)	14 (0.2)	70 (0.1)	55 (0.1)	401 (0.1)	296 (1.1)	2,746 (1.0)	1,173 (0.8)	8,458 (0.6)
Tetracycline	0 (0.0)	14 (0.0)	12 (0.0)	61 (0.0)	<5	8 (0.0)	8 (0.0)	55 (0.0)	757 (2.7)	6,568 (2.3)	3,907 (2.6)	29,312 (1.9)
Macrolides	500 (10.7)	4,105 (8.8)	3,941 (12.7)	29,882 (9.6)	452 (5.8)	4,172 (5.5)	5,747 (8.9)	46,933 (7.5)	7,161 (25.6)	69,856 (25.0)	40,321 (26.4)	364,409 (23.8)
Aminoquinolines	7 (0.1)	89 (0.2)	87 (0.3)	630 (0.2)	16 (0.2)	131 (0.2)	183 (0.3)	1,526 (0.2)	512 (1.8)	3,928 (1.4)	3,054 (2.0)	22,175 (1.5)
Amiodarone	47 (1.0)	325 (0.7)	464 (1.5)	3,528 (1.1)	57 (0.7)	503 (0.7)	997 (1.5)	8,058 (1.3)	107 (0.4)	991 (0.4)	873 (0.6)	7,650 (0.5)
Methoxypsoralen/PUVA	0 (0.0)	7 (0.0)	5 (0.0)	26 (0.0)	<5	<5	6 (0.0)	59 (0.0)	20 (0.1)	166 (0.1)	118 (0.1)	983 (0.1)
Other drug use, n (%)												
Aspirin	534 (11.5)	4,957 (10.6)	5,843 (18.8)	52,608 (16.9)	838 (10.7)	7,642 (10.1)	12,538 (19.5)	110,388 (17.6)	3,554 (12.7)	36,897 (13.2)	28,946 (18.9)	294,286 (19.3)
No-aspirin NSAID	2,354 (50.5)	20,932 (44.9)	16,807 (54.1)	141,560 (45.6)	3,452 (44.2)	32,538 (43.0)	37,193 (57.9)	325,814 (51.8)	14,316 (51.2)	144,555 (51.7)	84,588 (55.4)	825,266 (54.0)
Statins	1,057 (22.7)	9,487 (20.3)	9,818 (31.6)	87,152 (28.1)	1,739 (22.3)	15,600 (20.6)	22,833 (35.5)	203,655 (32.4)	4,242 (15.2)	43,233 (15.5)	31,442 (20.6)	316,126 (20.7)
Hydrochlorothiazide	NA	NA	NA	NA	NA	NA	NA	NA	2,571 (9.2)	22,533 (8.1)	18,444 (12.1)	164,335 (10.8)
Oral glucocorticoids	242 (5.2)	2,131 (4.6)	2,403 (7.7)	19,105 (6.1)	285 (3.6)	2,962 (3.9)	4,613 (7.2)	39,055 (6.2)	2,990 (10.7)	29,981 (10.7)	21,137 (13.8)	195,494 (12.8)
Charlson Comorbidity Index, n (%)												
0	NA	NA	NA	NA	6,897 (88.3)	66,667 (88.1)	56,047 (87.3)	550,046 (87.5)	23,682 (84.6)	230,460 (82.4)	120,076 (78.6)	1,181,335 (77.3)
1–2	NA	NA	NA	NA	740 (9.5)	7,349 (9.7)	6,596 (10.3)	63,691 (10.1)	3,844 (13.7)	43,899 (15.7)	29,015 (19.0)	304,957 (20.0)
≥3	NA	NA	NA	NA	172 (2.2)	1,683 (2.2)	1,587 (2.5)	15,030 (2.4)	452 (1.6)	5,421 (1.9)	3,730 (2.4)	41,918 (2.7)
Number of diagnoses,^2^ n (%)												
0	3,414 (73.2)	34,286 (73.6)	18,968 (61.1)	195,669 (63.0)	NA	NA	NA	NA	NA	NA	NA	NA
1	916 (19.6)	9,296 (19.9)	8,384 (27.0)	81,273 (26.2)	NA	NA	NA	NA	NA	NA	NA	NA
≥2	331 (7.1)	3,028 (6.5)	3,711 (11.9)	33,688 (10.8)	NA	NA	NA	NA	NA	NA	NA	NA
Diagnoses, n (%)												
Diabetes	552 (11.8)	5,362 (11.5)	5,124 (16.5)	50,752 (16.3)	893 (11.4)	9,039 (11.9)	12,295 (19.1)	117,248 (18.6)	1,474 (5.3)	16,560 (5.9)	9,563 (6.3)	116,582 (7.6)
COPD	346 (7.4)	3,710 (8.0)	3,160 (10.2)	30,656 (9.9)	382 (4.9)	4,146 (5.5)	6,483 (10.1)	58,354 (9.3)	630 (2.3)	9,010 (3.2)	6,187 (4.0)	69,724 (4.6)
Chronic kidney disease	178 (3.8)	1,237 (2.6)	2,173 (7.0)	15,543 (5.0)	195 (2.5)	1,751 (2.3)	3,728 (5.8)	29,450 (4.7)	200 (0.7)	1,979 (0.7)	1,481 (1.0)	14,324 (0.9)
Myocardial infarct	98 (2.1)	851 (1.8)	895 (2.9)	8,617 (2.8)	NA	NA	NA	NA	1,489 (5.3)	16,072 (5.7)	12,027 (7.9)	123,087 (8.1)
Smoking	998 (21.4)	8,967 (19.2)	6,088 (19.6)	52,212 (16.8)	1,243 (15.9)	13,214 (17.5)	8,921 (13.9)	82,914 (13.2)	NA	NA	NA	NA
Diagnoses associated with heavy alcohol consumption	NA	NA	NA	NA	144 (1.8)	2,036 (2.7)	1,841 (2.9)	18,827 (3.0)	342 (1.2)	6,185 (2.2)	2,714 (1.8)	36,130 (2.4)
Use of flecainide, n (%)												
Never-use	4,633 (99.4)	46,412 (99.6)	30,803 (99.2)	308,823 (99.4)	7,770 (99.5)	75,398 (99.6)	63,724 (99.2)	624,806 (99.4)	27,900 (99.7)	279,296 (99.8)	152,301 (99.6)	1,524,738 (99.7)
Ever-use	28 (0.6)	198 (0.4)	260 (0.8)	1,807 (0.9)	39 (0.5)	301 (0.4)	506 (0.8)	3,961 (0.6)	78 (0.3)	484 (0.2)	520 (0.3)	3,472 (0.2)
High-use (≥200,000 mg)	<5 (0.1)	27 (0.1)	50 (0.2)	247 (0.1)	5 (0.1)	44 (0.1)	90 (0.1)	690 (0.1)	37 (0.1)	183 (0.1)	185 (0.1)	1,295 (0.1)
Use of flecainide, cumulative dose, n (%)												
≤19 g	8 (0.2)	71 (0.1)	65 (0.2)	554 (0.2)	11 (0.1)	102 (0.1)	163 (0.2)	1297 (0.21)	15 (0.1)	101 (0.0)	108 (0.1)	731 (0.0)
20–99 g	11 (0.2)	76 (0.2)	91 (0.3)	701 (0.2)	11 (0.1)	94 (0.1)	157 (0.2)	1251 (0.2)	16 (0.1)	127 (0.0)	170 (0.1)	981 (0.1)
100–199 g	6 (0.1)	24 (0.1)	54 (0.2)	305 (0.1)	12 (0.1)	61 (0.1)	96 (0.1)	723 (0.1)	10 (0.0)	73 (0.0)	57 (0.0)	465 (0.0)
200–399 g	<5 (0.0)	22 (0.0)	39 (0.1)	188 (0.1)	<5	35 (0.0)	66 (0.1)	543 (0.1)	15 (0.1)	77 (0.0)	88 (0.1)	551 (0.0)
≥400 g	<5 (0.0)	5 (0.0)	11 (0.0)	59 (0.0)	<5	9 (0.0)	24 (0.0)	147 (0.0)	22 (0.1)	106 (0.0)	97 (0.1)	744 (0.0)

NA: not available.

^1^ Danish Health Registries include the Danish Cancer Registry, the National Prescription Registry, the National Patient Register, Registers in Statistics Denmark on educational level and income, and the Civil Registration System.

^2^ Diabetes, chronic obstructive pulmonary disease, chronic kidney disease, myocardial infarct, heart failure, peripheral vascular disease, cerebrovascular accident, dementia or Alzheimer’s disease, connective tissue disease, gastric ulcer, hemiplegia, and liver disease.

The association between the use of flecainide and the risk of melanoma and NMSC is reported in Table 2. Ever- and high-use of flecainide was associated with an increased risk of melanoma only in the Danish registries (adjusted OR of 1.58 (1.24–2.01) for ever-use; OR 1.97 (1.38–2.81) for high-use); however, there was no substantial variation in risk in analyses with cumulative flecainide dose as a categorical variable. Also, when restricting the analyses to ever-users of flecainide, the adjusted ORs for each 100 g increment in cumulative flecainide dose were inconclusive in all three databases [ORs of 1.04 (0.97–1.13) in Denmark, 0.96 (0.63–1.46) in BIFAP, and 1.05 (0.74–1.41) in SIDIAP].

**TABLE 2:** Association between exposure to flecainide and risk of skin cancer, according to cumulative amount of flecainide use.

	BIFAP		SIDIAP		Danish databases	
	Or (95 CI)^a^	Or (95 CI)^b^	Or (95 CI)^a^	Or (95 CI)^b^	Or (95 CI)^a^	Or (95 CI)^b^
Malignant melanoma						
Overall use of flecainide						
Non-use	1 (ref)	1 (ref)	1 (Ref.)	1 (Ref.)	1 (ref.)	1 (ref.)
Ever-use	1.32 (0.88–1.96)	1.22 (0.81–1.83)	1.28 (0.91–1.79)	1.23 (0.87–1.72)	1.61 (1.27–2.05)	1.58 (1.24–2.01)
High-use^c^	0.87 (0.26–2.86)	0.82 (0.25–2.73)	1.12 (0.44–2.82)	1.07 (0.42–2.71)	2.03 (1.42–2.89)	1.97 (1.38–2.81)
Cumulative dose of flecainide (mg)						
≤19	1.07 (0.51–2.23)	0.99 (0.47–2.08)	1.07 (0.57–1.99)	1.03 (0.55–1.93)	1.49 (0.86–2.56)	1.42 (0.83–2.46)
20–99	1.42 (0.75–2.68)	1.31 (0.69–2.49)	1.15 (0.62–2.15)	1.11 (0.59–2.08)	1.26 (0.75–2.12)	1.23 (0.73–2.07)
100–199	2.27 (0.92–5.58)	2.07 (0.83–5.13)	1.94 (1.05–3.61)	1.85 (0.99–3.45)	1.37 (0.71–2.66)	1.40 (0.72–2.72)
200–399	0.72 (0.17–3.05)	0.69 (0.16–2.96)	NA	NA	1.96 (1.12–3.40)	1.96 (1.12–3.43)
≥400	NA	NA	NA	NA	2.08 (1.31–3.29)	1.98 (1.24–3.14)
Dose–response (increment per 100 g of flecainide)	0.91 (0.62–1.33)	0.96 (0.63–1.46)	1.06 (0.76–1.40)	1.05 (0.74–1.41)	1.04 (0.97–1.11)	1.04 (0.97–1.13)
Non-melanoma skin cancer						
Overall use of flecainide						
Non-use	1 (ref)	1 (ref)	1 (Ref.)	1 (Ref.)	1 (ref.)	1 (ref.)
Ever-use	1.31 (1.15–1.49)	1.24 (1.09–1.42)	1.26 (1.15–1.38)	1.17 (1.06–1.28)	1.50 (1.37–1.65)	1.39 (1.26–1.52)
High-use	1.51 (1.12–2.06)	1.42 (1.04–1.93)	1.28 (1.02–1.59)	1.19 (0.95–1.48)	1.43 (1.23–1.67)	1.34 (1.15–1.56)
Cumulative dose of flecainide (mg)						
≤19	1.11 (0.85–1.43)	1.07 (0.82–1.38)	1.24 (1.06–1.47)	1.16 (0.99–1.37)	1.48 (1.21–1.81)	1.34 (1.10–1.65)
20–99	1.26 (1.01–1.57)	1.20 (0.96–1.50)	1.24 (1.05–1.46)	1.15 (0.97–1.36)	1.74 (1.48–2.04)	1.60 (1.36–1.89)
100–199	1.55 (1.16–2.07)	1.47 (1.10–1.97)	1.30 (1.05–1.61)	1.18 (0.96–1.47)	1.23 (0.93–1.62)	1.14 (0.87–1.50)
200–399	1.66 (1.17–2.35)	1.56 (1.10–2.20)	1.19 (0.92–1.53)	1.10 (0.85–1.42)	1.60 (1.28–2.00)	1.49 (1.19–1.87)
≥400	1.14 (0.60–2.18)	1.06 (0.56–2.03)	1.61 (1.05–2.49)	1.50 (0.97–2.31)	1.31 (1.06–1.61)	1.22 (0.99–1.51)
Dose–response (increment per 100 g of flecainide)	1.09 (0.99–1.20)	1.08 (0.98–1.19)	1.05 (0.97–1.13)	1.04 (0.96–1.13)	0.98 (0.95–1.01)	0.99 (0.96–1.02)

Compared with non-users, users of flecainide (ever- and high-use) were found to have a moderate increased risk of NMSC in all databases with adjusted ORs between 1.17 and 1.39 for ever-use and 1.19 and 1.42 for high-use. However, analyses by cumulative dose in the different databases showed no consistent dose–response patterns in associations. When restricting the analyses to ever-use of flecainide, the dose–response association was also not conclusive with ORs for each incremental cumulative dose increase of 100 g of flecainide were 0.99 (0.96–1.02) in Denmark, 1.08 (0.98–1.19) in BIFAP, and 1.04 (0.96–1.13) in SIDIAP.

The association between the use of flecainide and the risk of melanoma and NMSC stratified by age and sex was carried out with ever-use as exposure for the Spanish databases (Table 3) and with high-use as exposure for the Danish databases (Table 4). In general, the results stratified by age showed the highest estimates in the youngest age groups, but the precision was limited as reflected in the overlapping confidence intervals. When stratified by sex, all databases showed inconsistent results for both melanoma and NMSC; a higher risk of NMSC was found in male patients in the Danish databases, OR of 1.41 (1.16–1.70), and in female patients in the SIDIAP database, OR of 1.19 (1.05–1.36). In Spain, no association was observed between the ever-use of flecainide and the risk of melanoma stratified by sex, and a similar increased risk of melanoma was found in both male and female patients in Denmark.

**TABLE 3:** Association between ever-use of flecainide and risk of skin cancer, according to patient subgroups in the Spanish databases (SIDIAP and BIFAP). Reference: Never-users of flecainide in each subgroup (unexposed).

	BIFAP database			SIDIAP database		
Subgroup	Cases exposed/unexposed	Controls exposed/unexposed	Or (95 CI)^a^	Cases exposed/unexposed	Controls exposed/unexposed	Or (95% CI)^b^
Malignant melanoma						
<50 years	<5/1,285	11/12841	NA	11/23748	<5/2,511	NANA
50–59 years	<5/871	18/8730	NA	34/14351	<5/1,484	NA
60–74 years	15/1,442	92/14479	1.51 (0.85–2.67)	128/24408	22/2453	1.61 (1.01–2.57)
75 + years	9/1,035	77/10362	0.94 (0.46–1.92)	128/12891	15/1,322	1.16 (0.67–2.00)
Male	17/2,041	97/20483	1.45 (0.85–2.50)	144/33281	17/3424	1.11 (0.67–1.86)
Female	11/2,592	101/25929	0.97 (0.51–1.84)	157/42117	22/4346	1.32 (0.83–2.08)
Non-melanoma skin cancer (Total)						
<50 years	<5/2,489	14/24,911	NA	34/60564	6/6340	1.44 (0.60–3.49)
50–59 years	9/3,724	82/37253	1.00 (0.50–2.01)	149/79921	20/8137	1.14 (0.70–1.85)
60–74 years	88/10,334	620/103609	1.18 (0.94–1.49)	1611/244957	190/24772	1.06 (0.91–1.24)
75 + years	159/14,256	1091/143050	1.28 (1.08–1.51)	2167/239364	290/24475	1.23 (1.09–1.40)
Male	130/14,895	860/149,390	1.29 (1.07–1.56)	1886/300386	235/30,584	1.14 (0.99–1.31)
Female	130/15,908	947/159,433	1.20 (1.00–1.45)	2075/324420	271/33,140	1.19 (1.05–1.36)

CI, confidence interval; NA, not applicable; OR, odds ratio.

^a^ Adjusted for age, gender (by risk-set matching and the conditional analysis), and time up to index date since first day of register in the database and additionally for 1) use of photosensitizing drugs: topical retinoids, oral retinoids, tetracycline, macrolides, aminoquinolines, amiodarone, and methoxypsoralen; 2) use of other drugs: aspirin, non-aspirin non-steroidal anti-inflammatory drugs, statins, and glucocorticoids; 3) comorbidity: diabetes, chronic obstructive pulmonary disease, chronic kidney disease, myocardial infarct, heart failure, peripheral vascular disease, cerebrovascular accident, dementia or Alzheimer’s disease, connective tissue disease, gastric ulcer, hemiplegia, and liver disease; and 4) smoking.

^b^ Adjusted for age, gender (by risk-set matching and the conditional analysis), and time up to index date since first day of register in the database and additionally for 1) use of photosensitizing drugs: topical retinoids, oral retinoids, tetracycline, macrolides, aminoquinolines, amiodarone, and methoxypsoralen; 2) use of other drugs: aspirin, non-aspirin non-steroidal anti-inflammatory drugs, statins, and glucocorticoids; 3) comorbidity: diabetes, chronic obstructive pulmonary disease, and chronic kidney disease; 4) smoking; and 5) Charlson Comorbidity Index.

**TABLE 4:** Association between high-use of flecainide and risk of skin cancer, according to patient subgroups in the Danish databases. Reference: Never-users of flecainide in each subgroup (unexposed).

Subgroup	Cases exposed/unexposed	Controls exposed/unexposed	Or (95% CI)^a^
Malignant melanoma			
<50 years	<5	<5	NA
50–59 years	<5	-*/53793	NA
60–74 years	22/9,418	96/94354	2.34 (1.46–3.74)
75 + years	13/4,110	65/41203	1.80 (0.99–3.29)
Male	23/12,789	115/128122	1.96 (1.25–3.09)
Female	14/15,111	68/151174	1.98 (1.11–3.54)
Non-melanoma skin cancer (Total)			
<50 years	—	9/197,433	NA
50–59 years	5/26,549	72/265523	0.73 (0.29–1.81)
60–74 years	110/66,986	664/671005	1.57 (1.28–1.92)
75 + years	70/39,024	550/390777	1.15 (0.90–1.48)
Male	126/74,159	832/742790	1.41 (1.16–1.70)
Female	59/78,142	463/781984	1.20 (0.91–1.57)

CI, confidence interval; NA, not applicable; OR, odds ratio.

^a^Adjusted for age, gender (by risk-set matching and the conditional analysis), and time up to index date since the first day of register in the database and additionally for 1) use of photosensitizing drugs: topical retinoids, oral retinoids, tetracycline, macrolides, aminoquinolines, amiodarone, and methoxypsoralen; 2) use of other drugs: aspirin, non-aspirin non-steroidal anti-inflammatory drugs, statins, and glucocorticoids; 3) comorbidity: diabetes, chronic obstructive pulmonary disease, and chronic kidney disease; and 4) Charlson Comorbidity Index.

* not shown to preserve anonymity for counts n < 5.

## Discussion

In this multi-database population-based case–control study, we found an association between the use of flecainide and risk of melanoma (Denmark only) and NMSC (Denmark and Spain), but with no clear dose–response pattern in any of the analyses.

Although many antiarrhythmics are known to have photosensitizing properties and photosensitivity has been related to an increased risk of skin cancers (Robinson et al., 2013), the few studies that have assessed the associated risk of skin cancer with the intake of these drugs found conflictive results; while case reports support an increased risk of NMSC associated with amiodarone (Monk, 1995; Hall et al., 2004; Maoz et al., 2009), two large observational studies from Taiwan and Denmark found no association between the use of amiodarone and the overall risk of skin cancer (Su et al., 2013; Rasmussen et al., 2020). No previous study has analyzed the risk of melanoma and NMSC associated with the intake of other antiarrhythmics, such as flecainide, nor has it analyzed the risk according to the cumulative exposure of the drug.

The increased melanoma risk found in Denmark (up to 2-fold among high-users of flecainide), but not in Spain; in the current study, it could be explained by differences in environmental factors (i.e., UV exposure) and genetic and phenotypic differences (e.g., skin pigmentation) as such factors would modify the proposed photosensitizing effects of flecainide (ECIS, 2021). Thus, the skin pigmentation difference, with the Danish having fairer skin compared with the Spanish, could make them more prone to sunburn at lower sunlight exposure levels, increasing the risk of melanoma (Tuohimaa et al., 2007). This increased risk could be further accentuated with the use of a photosensitizing drug such as flecainide, partly explaining our results. Conversely, it has been suggested that NMSC is more related to the cumulative sunlight exposure, rather than to episodic sunburns, which could be increased by the use of flecainide and could explain the overall risk found in both countries.

When analyzing the cumulative exposure of flecainide, an association was observed between doses ≥200 mg and melanoma in Denmark. Although the association found was statistically significant, the clinical implications of it are not clear; there is no consistent pattern with increasing cumulative doses of flecainide. Similarly, the dose–response patterns were not apparent for NMSC which may indicate the existence of unmeasured confounders. One of these could be the socioeconomic status (SES); high SES has been associated both with a higher risk of being diagnosed with skin cancer (Doherty et al., 2010) and a greater likelihood of being prescribed an antiarrhythmic drug (Eberly et al., 2021). In this study, although the information related to SES was available for SIDIAP (through the MEDEA deprivation index) and the Danish registries (through the level of education) (data not shown), the lack of uniformity in the data prevented us to adjust for it. In SIDIAP, a higher proportion of skin cancer (19.3% of melanoma and 16.2% of NMSC) was observed among the least deprived compared with the most deprived (9.9% of melanoma and 8.0% of NMSC). Similarly, in Denmark, a higher proportion of skin cancer (74.5% of melanoma and 68.6% of NMSC) was observed among those who had achieved a medium/higher education compared with those who had achieved a short education (24.1% for melanoma and 29.3% for NMSC). This suggests that socioeconomic status could have influenced our results.

The main advantage of the study is the multinational approach, investigating the association between the use of flecainide and non-melanoma and melanoma skin cancer in two countries in which different ethnicities/skin types and different UV exposure patterns can be assumed, and with potential different utilization patterns of flecainide. Furthermore, data from the nationwide Danish health registries, SIDIAP, and BIFAP are generally considered to be of high validity and well suited for epidemiological research. The main limitation of the study is the lack of direct or indirect measures of UV exposure, as well as the lack of uniformity gathering the socioeconomic status which prevented us to adjust to it. There is no reason to suspect that users of flecainide will differ substantially from the background population regarding sun exposure habits and the likely impact of this factor is thus expected to be limited within each database. Underestimation of the incidence of skin cancer in primary care databases is also possible which could have contributed to the underestimation of the cases. Other potential limitation in BIFAP is the use of prescription information for drug exposure when drug dispensation was not available which could have overestimated our intake of flecainide. However, given that we found similar results compared with the other Spanish database (SIDIAP) in where only dispensations were collected, the effect of this overestimation is likely to be minimal. At last, information on histology or localization was not examined.

## Conclusion

Flecainide use was associated with an increased risk of melanoma (Denmark only) and NMSC, but without substantial evidence of dose–response patterns. In Spain, flecainide use was associated with an increased risk of NMSC with no dose–response pattern. Further studies are needed assessing for possible unmeasured confounders.

## Data Availability

Data partners contributing to this study remain custodians of their individual patient-level health information. Due to data protection regulations, individual level data from this study and administrative registries cannot be shared by the authors. Data access can be granted to Danish scientific and university-based organizations after application to Statistics Denmark (www.sundhedsdatastyrelsen.dk). According to the Spanish law and the IDIAP JGol Ethical Review Board, it is prohibited to publicly share data with personal information. Requests to access the Spanish datasets should be directed to https://www.sidiap.org/index.php/solicituds for the SIDIAP database and to www.bifap.org for the BIFAP database.
