# Supplementary material for: Flecainide and risk of skin neoplasms: Results of a large nested case–control study in Spain and Denmark
Source: Front Pharmacol. 2022 Dec 22;13:1002451. doi: 10.3389/fphar.2022.1002451 (PMC9822716; doi:10.3389/fphar.2022.1002451)

Supplementary Material

| **Supplementary Table 1** Diagnoses (International Classification of Disease (ICD-10) and drug codes (WHO Anatomical Therapeutic Chemical (ATC)) included | | |
| --- | --- | --- |
| **Outcomes** | | |
| Non-Melanoma Skin Cancer | *ICD-10* | C44.0-C44.9 |
| Malignant melanoma | *ICD-10* | C43.0-C43.9 |
| **Exposure** | | |
| Flecainide | *ATC code* | C01BC04 |
| **Exclusion criteria** | | |
| Any cancer (except non-melanoma skin cancer) | *ICD-10* | C00-97 |
| Organ transplant | *ICD-10* | Z94 (except Z94.5 and Z94.7) |
|  | Nordic Classification of Surgical Procedures *-code* | KFQA, KFQB, KGDG, KJJC, KJLE, and KKAS |
| HIV | *ICD-10* | B20-24 and Z21 |
| Azathioprine | *ATC code* | L04AX01 |
| Cyclosporine | *ATC code* | L04AD01 |
| Mycophenolate mofetil | *ATC code* | L04AA06 |
| **Photosensitizing drugs** | | |
| Topical retinoids | *ATC code* | D10AD |
| Oral retinoids | *ATC code* | D05BB D10BA01 |
| Tetracycline | *ATC code* | J01AA07 |
| Macrolides | *ATC code* | J01FA |
| Aminoquinolines | *ATC code* | P01BA |
| Amiodarone | *ATC code* | C01BD01 |
| Methoxypsoralen | *ATC code* | D05AD, D05BA |
| PUVA (Psoralen and Ultraviolet A phototherapy) | *ICD-10* | BNGA1 |
| **Other drugs** | | |
| Aspirin | *ATC code* | B01AC06, B01AC30, N02BA01, and N02BA51 |
| Non-aspirin NSAIDs | *ATC code* | M01A excl. M01AX |
| Statins | *ATC code* | C10AA |
| Hydrochlorothiazide | *ATC code* | C03AA03, C03AB03, C03AX01, C09DX03, C03EA, C09BA, C09DA, C07B, C09DX01, C09XA52, C09XA54 (excluded: C07BA06, C09BA04) |
| Oral glucocorticoids | *ATC code* | H02AB |
| **Diagnoses (diagnostic code or drug marker)** | | |
|  |  |  |
| Diagnosis and codes associated with heavy alcohol consumption | *ICD-10* | G31.2, G62.1, G72.1, I42.6, F10.2, K70, K86.0 |
|  | *ATC code* | N07BB |
| Diabetes | *ICD-8* | 249.00, 249.09, 250.00, 250.09 |
|  | *ICD-10* | E10-E14 |
|  | *ATC code* | A10 |
| COPD | *ICD-8* | 490.00 491.00 491.01 491.03 |
|  | *ICD-10* | J42-J44 |
|  | *ATC code* | R03BB |
| Chronic kidney disease | *ICD-10* | E102, E112, E122, E132, E142, I12 (÷I129), N01, N03, N083, N085, N118C, N14, N150, N16 (÷ N160), N18 (÷N181), N19, N26, P960, Q601, Q602, Z992 |
|  |  |  |
| Charlson comorbidity index* | *Weight* | ICD-10 code |
| Myocardial infarction | 1 | I21.x, I22.x, I25.2 |
| Congestive heart failure | 1 | I09.9, I11.0, I13.0, I13.2, I25.5, I42.0, I42.5–I42.9, I43.x, I50.x, P29.0 |
| Peripheral vascular disease | 1 | I70.x, I71.x, I73.1, I73.8, I73.9, I77.1, I79.0, I79.2, K55.1, K55.8, K55.9, Z95.8, Z95.9 |
| Cerebrovascular disease | 1 | G45.x, G46.x, H34.0, I60.x–I69.x |
| Dementia | 1 | F00.x–F03.x, F05.1, G30.x, G31.1 |
| Chronic pulmonary disease | 1 | I27.8, I27.9, J40.x–J47.x, J60.x–J67.x, J68.4, J70.1, J70.3 |
| Rheumatic disease | 1 | M05.x, M06.x, M31.5, M32.x–M34.x, M35.1, M35.3, M36.0 |
| Peptic ulcer disease | 1 | K25.x–K28.x |
| Mild liver disease | 1 | B18.x, K70.0–K70.3, K70.9, K71.3–K71.5, K71.7, K73.x, K74.x, K76.0, K76.2–K76.4, K76.8, K76.9, Z94.4 |
| Diabetes without chronic complication | 1 | E10.0, E10.1, E10.6, E10.8, E10.9, E11.0, E11.1, E11.6, E11.8, E11.9, E12.0, E12.1, E12.6, E12.8, E12.9, E13.0, E13.1, E13.6, E13.8, E13.9, E14.0, E14.1, E14.6, E14.8, E14.9 |
| Diabetes with chronic complication | 2 | E10.2–E10.5, E10.7, E11.2–E11.5, E11.7, E12.2–E12.5, E12.7, E13.2– E13.5, E13.7, E14.2–E14.5, E14.7 |
| Hemiplegia or paraplegia | 2 | G04.1, G11.4, G80.1, G80.2, G81.x, G82.x, G83.0–G83.4, G83.9 |
| Renal disease | 2 | I12.0, I13.1, N03.2–N03.7, N05.2– N05.7, N18.x, N19.x, N25.0, Z49.0–Z49.2, Z94.0, Z99.2 |
| Moderate or severe liver disease | 3 | I85.0, I85.9, I86.4, I98.2, K70.4, K71.1, K72.1, K72.9, K76.5, K76.6, K76.7 |
| *The same comorbidities with different severity were mutually exclusive: diabetes with chronic complications and diabetes without chronic complications and mild liver disease and moderate or severe liver disease. | | |


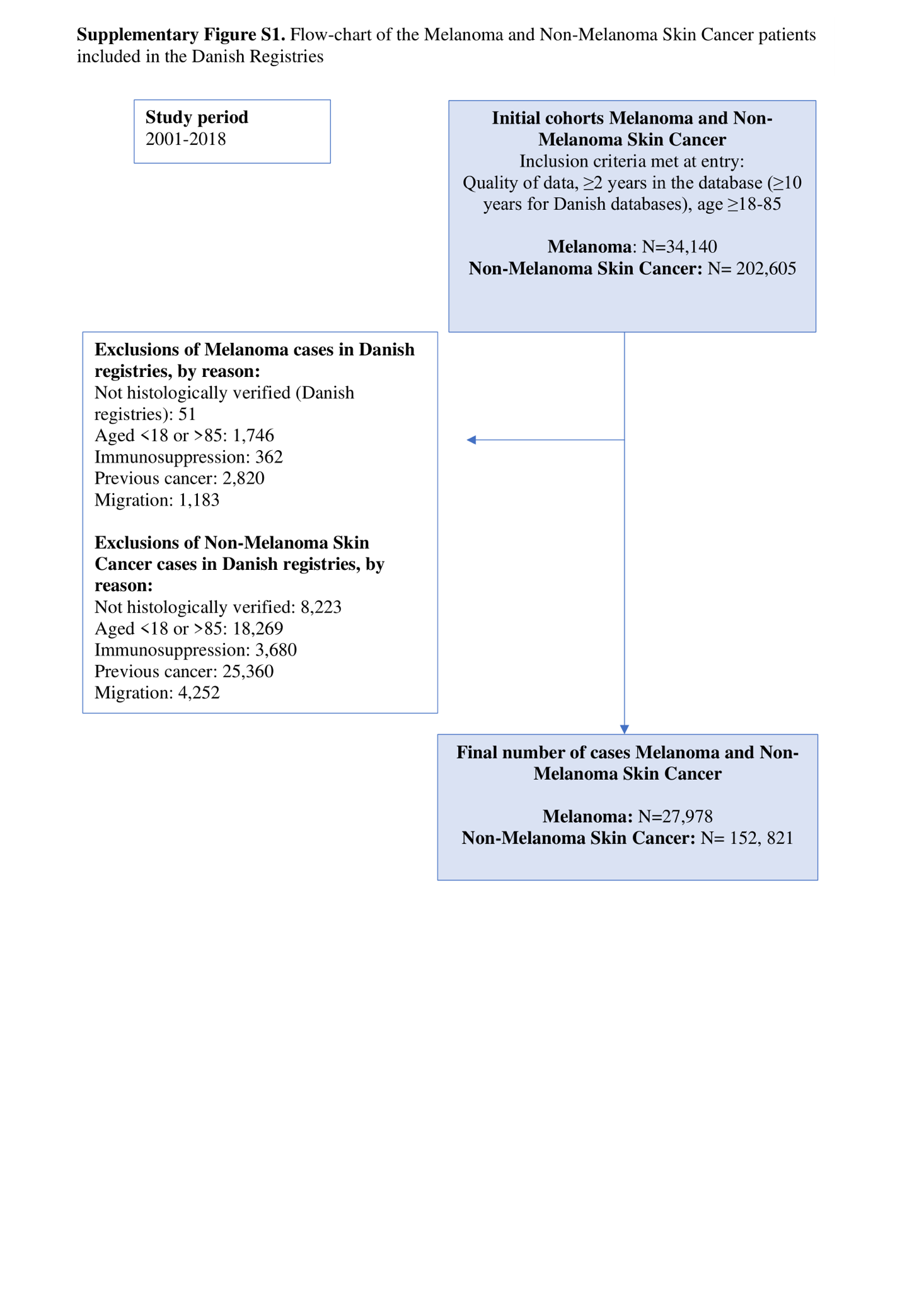


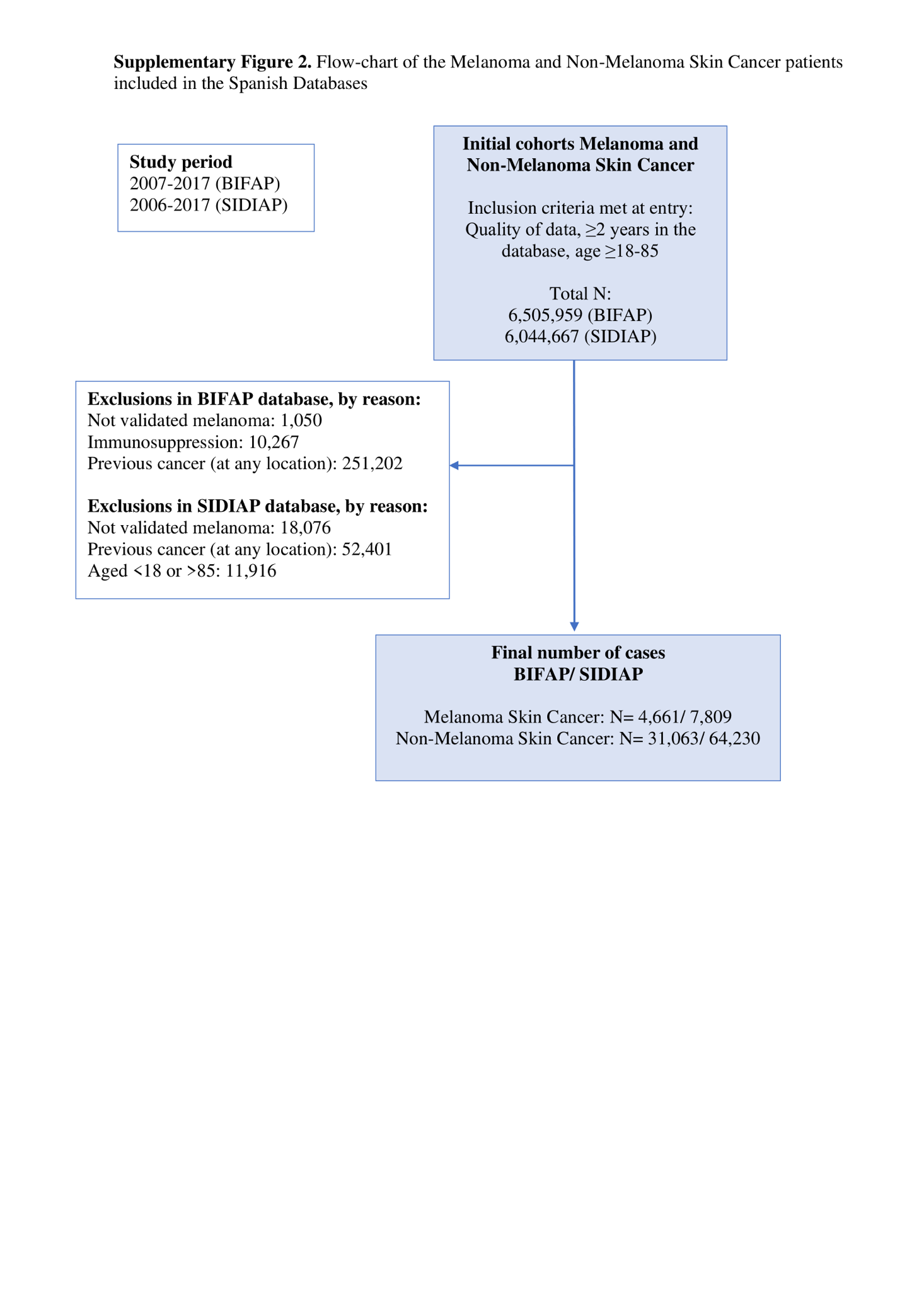

Supplement: Supplementary file 1 [file Table1.DOCX]
